# Pneumorrhachis Secondary to an Infected Sacral Decubitus Ulcer

**DOI:** 10.7759/cureus.17502

**Published:** 2021-08-27

**Authors:** Olinda Lima Miranda, Anabela Carvalho, Angela Almeida, Magda Fernandes, Jorge Cotter

**Affiliations:** 1 Internal Medicine, Hospital Senhora da Oliveira, Guimarães, PRT

**Keywords:** pneumorrhachis, air spinal canal, endochannel emphysema, decubitus ulcer, coma

## Abstract

Pneumorrhachis (PR) is a rare phenomenon, which consists in the presence of air in the spinal canal. There are various aetiologies, being the most common traumatic, non-traumatic and iatrogenic. The diagnosis is primarily done through radiographic findings and it is necessary to understand the mechanism behind its origin. PR secondary to decubitus ulcer (DU) infection is rare. PR is associated with great morbidity and mortality. In selected cases, surgical intervention may be necessary.

A 67-year-old woman, dependent, was admitted to the emergency room (ER) and diagnosed with an infected sacral DU, later discharged with antibiotics. She was readmitted to the ER two weeks later, with prostration and fever. On examination, she scored five points on the Glasgow coma scale, had bilateral Babinsky sign and a deep sacral ulcer with bone exposure. A cranial computerized tomography (CT) demonstrated “high cervical and endochannel emphysema in the upper slope of the cervical segment” and the CT scan of the spine showed “endochannel air along the cervical-dorsal and lumbar rachis in an epidural location and inside the dural sac (evoking laceration of the dura mater) (…) and densification of the sacrococcygeal soft tissues (diagnosis of PR secondary to DU infection)”. Broad-spectrum antibiotics were started and the patient was evaluated by General Surgery, which described a large sacral ulcer with signs of the previous debridement and bone exposure, with no indication for surgical debridement, only for chemical debridement. Despite all the measures instituted, the patient died in the ER.

## Introduction

Pneumorrhachis (PR) is a rare phenomenon that consists of the presence of air in the spinal canal. The air can be present in the entire length of the spinal canal, from the lumbar to the cervical region, or only in the region of dissection/injury. Being so rare, it is important to understand the mechanisms and pathologies involved in its origin [1-3].

PR may be of iatrogenic, traumatic and non-traumatic aetiology [1,4,5,6]. In some cases it is not possible to define the mechanism behind its origin [1].

As to aetiology, we have iatrogenic manipulation during surgical, diagnostic (lumbar puncture) and anesthetic procedures, traumatic or penetrating injuries of the spine, cancer and spontaneous [1]. There are some cases of PR associated with infectious diseases, such as epidural abscesses, hematogenous dissemination, intraperitoneal sepsis and complications of decubitus ulcer [2,3,7,8]. The involvement and production of gas by gangrene in the spinal canal is extremely rare [9].

PR is normally asymptomatic and therefore the diagnosis is mainly imagiological [1,6,7]. Radiography is useful as a primary exam because it allows the visualization of a large quantity of air in the spine. The gold standard exam is computerized tomography (CT), since it detects intra- and extra-dural air and allows the diagnosis of other traumatic injuries or mechanisms that cause PR. Magnetic resonance imaging or myelography are more sensitive exams to detect lesions and determine differential diagnosis [1].

PR is associated with high mortality and morbidity. There are no guidelines for the treatment of PR due to its rarity [2].

There may be a loss of cerebrospinal fluid in PR, leading to a decrease of intracranial pressure and surgical intervention for its correction may be needed [1,3]. In turn, when air enters the intracranial compartment there is an increase of intracranial pressure, which is a surgical emergency [1]. The use of antibiotics as prophylactic therapy for meningitis is not established and is controversial [1,4,5].

The causes of PR should be identified and treated accordingly.

## Case presentation

A 67-year-old female, dependent on her daily activities, with a history of Alzheimer’s disease, type 2 diabetes mellitus, arterial hypertension and dyslipidemia was observed in the ER two weeks before admission and diagnosed with an infected sacral decubitus ulcer and was discharged with antibiotic (cefolosporin). Two weeks later she was admitted in the ER with prostration with one week of evolution and fever with four days evolution, which resolved with paracetamol. On admission, she was apyretic, normotensive, tachycardic, hyperglycemic, scored five points on the Glasgow Coma Scale, had bilateral Babinsky sign and a deep sacral ulcer with bone exposure. Analytically she presented leukocytosis 15,200/uL, with neutrophilia 93%, glucose 469 mg/dL, urea 58 mg/dL, creatinine 1,30 mg/dL, sodium 153 mEq/L, albumin 2,1 g/dL, C-reactive protein 517mg/L and blood cultures (4 samples) which posteriorly isolated a gram-positive *coccos* - *Streptococcus anginosus*.

**Figure 1:**
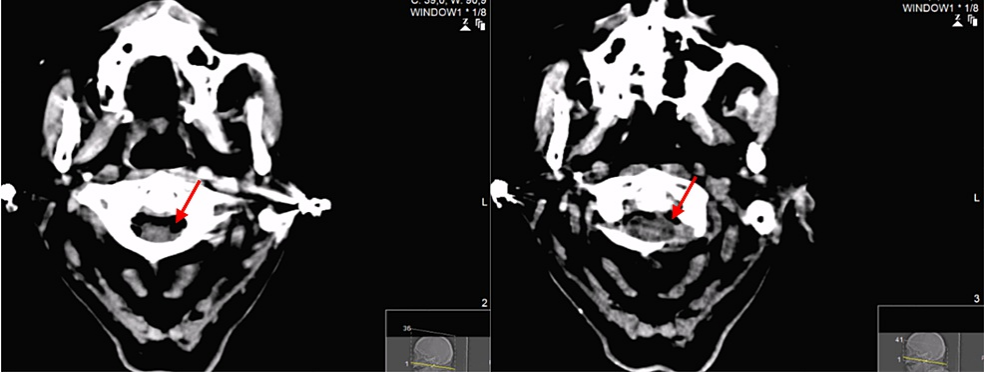
Endochannel cervical emphysema

A cranial CT demonstrated “high cervical and endochannel emphysema in the upper segment of the cervical rachis (…) (Figure 1).”

**Figure 2:**
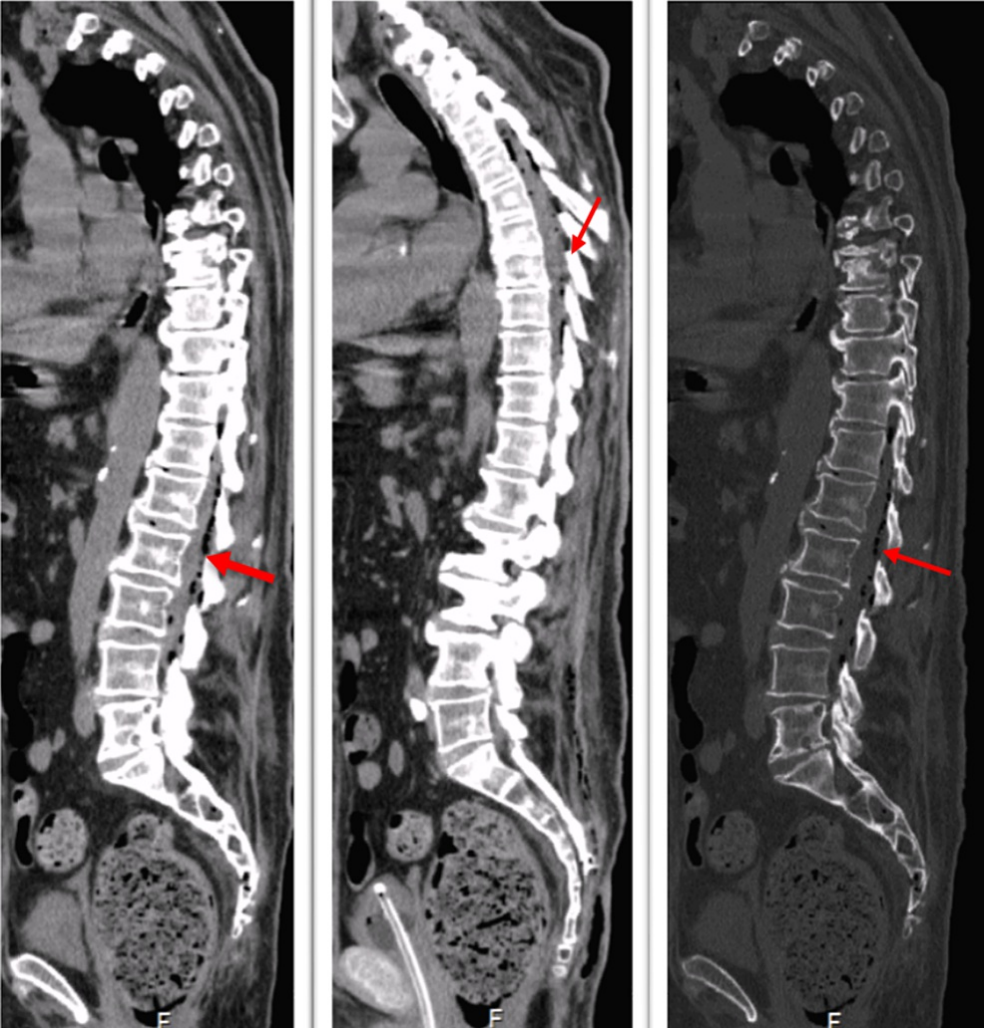
Endochannel air through the cervical-dorsal and lumbar rachis in epidural location and interior of the dural sac

A spine CT was performed showing “the presence of endochannel air along the cervical-dorsal and lumbar rachis in an epidural location and inside the dural sac (evoking laceration of the dura mater), apparently insufficient to cause spinal cord compression; as well as air bubbles in the paraespinal soft tissues of the lumbar region and “densification” of the sacrococcygeal soft tissues muscles (possible infection with origen in the ulcerated lesion) (Figure 2), showing the presence of air with compression of the right pre-sacral region and homolateral psoas muscle. Small round bone erosions on the right vertebral platforms of L4-L5 and L5-S1, may translate a mixture of degenerative alterations and the initial fase of infectious spondylodiscal”.

Broad-spectrum antibiotics were initiated.

She was evaluated by General Surgery, which described a large sacral ulcer, with signs of the previous debridement and sacral bone exposure, without indication for surgical debridement, only chemical debridement.

Despite all the measures instituted, the patient died in the ER.

## Discussion

Infectious pneumorraquis can be secondary to hematogenous dissemination or extension of a local infectious process, such as vertebral osteomyelitis, caused by gas-producing microorganisms [2]. PR secondary to decubitus ulcer is rare, but there are some cases described in the literature, being the initial clinical manifestation of a headache and may evolve to changes in the state of consciousness and meningitis [3]. George R. Thompson, et al. described a case of pneumorraquis associated with a bacterial infection with gas production starting with a decubitus ulcer, in which two agents were isolated in the blood cultures, *Clostridium subterminale* and *Streptococcus anginosus *[8]. In the case presented, the origin of the pneumorraquis was also a decubitus ulcer, associated with bacteremia from *Streptococcus anginosus*, with gas production in the surrounding tissues and structures.

Given the rarity of PR, a high degree of suspicion is important for diagnosis. The exam of choice is CT and the treatment depends on the type of lesion that led to the PR and the clinical manifestation. In some cases, surgical treatment is necessary [1,3].

## Conclusions

Infectious PR is extremely rare. The treatment of decubitus ulcers in regions overlapping the spine should not be neglected, given the possibility of infection by gas-producing microorganisms affecting the spinal canal. When an infectious PR is suspected, broad-spectrum antibiotics should be empirically initiated. PR is mostly asymptomatic, but when symptomatic it is associated with high morbidity and mortality, which was the outcome of this case.
